# A minority among Chinese psychiatrists: the current state of development of child and adolescent psychiatrists

**DOI:** 10.3389/fpsyt.2025.1646657

**Published:** 2025-07-24

**Authors:** Zhiwei Liu, Shu Cui, Kun Liu, Gaofeng Yao, Yun Liu, Huanzhong Liu

**Affiliations:** ^1^ Department of Psychiatry, The Third People’s Hospital of Fuyang, Fuyang, China; ^2^ Department of Psychiatry, Chaohu Hospital of Anhui Medical University, Hefei, China; ^3^ School of Mental Health and Psychological Sciences, Anhui Medical University, Hefei, China

**Keywords:** child, China, minority groups, psychiatrists, adolescent

## 1 Introduction

At the beginning of 2025, the National Health Commission (NHC) of China designated 2025–2027 as the “Years of Pediatric and Mental Health Services,” aiming to use the next three years to continuously expand the professional workforce, enhance capabilities, improve standards, and optimize services, thereby addressing the shortcomings in pediatric and mental health care. According to the Global Burden of Disease Study 2019, mental disorders remain one of the top ten leading causes of disease burden globally (1). According to the China Statistical Yearbook 2024, China has a population of 230 million children aged 0-14 (2). The overall prevalence of mental disorders among the 6–16 age group nationwide was 17.5% (3), indicating that at least 40 million patients in China require child and adolescent mental health services. The latest systematic review revealed that mental disorders accounted for the most significant proportion of years of healthy life loss (YLDs) in assessments of children and adolescents’ health in China (4). Additionally, research showed that throughout the life course, 25% of YLDs attributable to mental disorders are recorded during adolescence and early adulthood (5).

## 2 Shortage of child and adolescent psychiatrists

There is an extreme shortage of CAPs in China. Literature indicates that China began CAP training as early as 1975; however, as of 2019, the number of CAP participants had not exceeded 500 (6). A study indicated that in China, there are only 0.09 cases of CAPs per 100,000 children aged 14 and under. In contrast, this figure is 3.95 in Singapore, 1.97 in Japan, 11.29 in Israel, and 1.57 in Thailand, all of which were higher than that of China (7). Furthermore, there are approximately 8,300 CAPs in the United States. For every 100,000 children aged 14 and under, there are about 13.6 CAPs (8). In 2019, a Correspondence in the Lancet Psychiatry highlighted this situation (9); due to the authority of its source, this figure has been widely quoted in both official and non-official media. Nevertheless, as there is currently no specialized board-examination for the qualification of CAPs in China, it is difficult to accurately count the exact number of CAPs. Current evidence suggests that the number of CAPs has not significantly increased since 2019, and it remains challenging to provide timely and effective mental health services to a large number of children and adolescents (10).

As of 2020, the majority of doctors responsible for diagnosing and treating mental disorders in children and adolescents were psychologists or adult psychiatrists, with CAPs accounting for only 10% (6). Even among this small number of CAPs, there are still physicians who have not undergone systematic training, as there are currently few CAP training institutions in China and no officially recognized CAP certification system (11). In China, child mental health professionals work within a four-tier system. This system encompasses community health centers, county - and municipal-level medical institutions, provincial mental health centers, and the highest level—national mental health centers and national maternal and child health centers—with a trend toward a greater concentration of professionals at higher-level institutions (12). As a result, most CAPs are concentrated in large and medium-sized cities, while smaller cities (and even some provincial capitals) lack CAPs. A study in JAMA Psychiatry showed that in areas with insufficient mental health resources, the suicide rate among adolescents had significantly increased (the adjusted incidence rate ratio was 1.16) (13). Furthermore, the scores of areas with shortage of mental health resources were rated from 0 to 25 points (the higher the score, the more severe the labor shortage). The results showed that for every 1-point increase in the rated score, the adjusted adolescent suicide rate increased by 4%, indicating that the severity of human resource shortage was positively correlated with the increased risk of suicide (13). Thus, the scarcity of mental health personnel may lead to severe adverse consequences.

## 3 Status of mental health resources in China

To address the shortage of mental health service capacity, the Chinese government has introduced a series of policies and regulations to promote mental health, including undergraduate and graduate education in psychiatry, a national standardized training program for psychiatrists, and a certification program for internal medicine physicians to obtain psychiatric licenses (14). According to the “China Health and Wellness Statistical Yearbook 2023”, by the end of 2022, there were a total of 2,277 mental hospitals across the country, with a total of 840,871 beds (accounting for 8.62% of all beds in medical and health institutions), and a total of 56,166 registered practicing (assistant) psychiatrists, among whom 50,005 were practicing psychiatrists, accounting for 1.7% of the total number of physicians in the country. There are 33 mental health prevention and treatment institutions (stations, centers) nationwide, with 314 registered practicing psychiatrists (15). As of 2022, the number of registered psychiatric nurses across the country was 146,451, an increase of approximately 5% compared to 2020 (15, 16). In the early 1990s, the Psychiatric Nursing Professional Committee of the Chinese Nursing Association was established in China, providing a solid backing for the professional scope, industry standards and high-quality development of psychiatric nursing. Geographically speaking, the total number of open psychiatric beds and practicing (assistant) psychiatrists and nurses in the eastern region is significantly higher than that in the central and western regions (16). The quantity of mental health resources per unit land area in the western region is significantly lower than that in the eastern and central regions, showing a close correlation among the degree of population density, the level of economic development and the level of mental health resources.

Although the number of psychiatric hospitals, the number of psychiatrists (including assistant psychiatrists), and the annual number of outpatient visits for mental health services have been steadily increasing (17). However, among the tens of thousands of psychiatrists in China, only a few hundred are specialized in child and adolescent psychiatry, accounting for a small proportion of the total. Additionally, literature indicated that among mental health institutions nationwide in China, only 0.53% (312/5936) have pediatric psychiatric wards (16), and pediatric psychiatric beds account for less than 1% of the total number of psychiatric beds (18). In China, a minor refers to a natural person who is under the age of eighteen. However, there is a view that the age range of 10 to 24 is more suitable for the development of today’s teenagers. Because the more mature development of emotional regulation, social relationships and executive functions during adolescence may continue until one’s twenties (19). Basically, all child psychiatry wards in mental health specialized hospitals admit people under the age of 18. The age range of the population admitted to a relatively small number of child psychiatric wards has been expanded to 24 years old. However, this diagnosis and treatment model has no legal basis and has not been recognized nationwide. In summary, the current shortages of CAP personnel and pediatric psychiatric beds, among other related issues, require urgent attention.

### 3.1 Undergraduate and graduate education in psychiatry

As physical health and nutrition issues have mainly been addressed in most regions of China, mental health has become one of the core health concerns for the general population. The field of mental health in China has developed relatively late and lags significantly behind. In recent years, China’s higher education system has supported colleges and universities in expanding enrollment in mental health-related programs, including psychiatry, psychological rehabilitation therapy, and psychology. It has also encouraged colleges and universities with the necessary resources to establish mental health-related programs, guided more outstanding high school graduates to apply for such programs, and incorporated professional courses in psychiatry into the curriculum for cultivating medical talent. However, a report noted that fewer than 2,000 undergraduate students are admitted to psychiatry programs nationwide each year (20). Additionally, due to poor working conditions, low income, and low social status, over half of undergraduates do not pursue careers in psychiatry (21). In addition to a shortage of psychiatrists, clinical psychotherapists also struggle to meet actual demand. According to statistics from the Chinese Mental Health Association, there are currently only 6,000 to 8,000 psychotherapists in China and fewer than 30,000 psychological counselors capable of providing counseling services (22). In mental health medical institutions, psychiatrists account for as high as 66% of the personnel providing psychological treatment/counseling services, indicating that in addition to their primary duties, some psychiatrists also concurrently serve as clinical psychotherapists, further exacerbating the shortage of clinical medical resources (23). Therefore, expanding undergraduate education in psychiatry, psychological rehabilitation therapy, or psychology remains a beneficial measure to increase the number of mental health professionals.

### 3.2 Standardized training for psychiatric residents

Since 2014, to address the urgent need for training psychiatric doctors, standardized training for psychiatric residents has been gradually promoted in China. The latest training objectives clearly outline six core competencies: professional ethics, professional skills, patient management, communication and collaboration, teaching abilities, and continuous learning and improvement (24). As of 2023, there are a total of 217 psychiatric specialty training bases nationwide, including 190 general hospitals and 27 psychiatric specialty hospitals (24). Due to the need for professional title promotion within the medical system, nearly all newly registered psychiatrists over the past decade have been required to undergo standardized residency training in psychiatry to develop further the necessary professional independence and standardized clinical diagnostic and treatment capabilities.

Due to the limited number of pediatric psychiatric wards, most of which are concentrated in major cities such as Beijing, Shanghai, and Guangzhou, many psychiatrists may find it challenging to engage in clinical practice related to child and adolescent mental disorders during their training period (11). After obtaining the title of attending physician, psychiatrists may continue to participate in specialized CAP training programs at higher-level training institutions, with the recommendation of their institutions. Training institutions in Beijing, Shanghai, Jiangsu, and Zhejiang provinces play a dominant role (6). However, even among psychiatrists who participate in CAP training programs, the proportion engaged in child and adolescent psychiatry remains below 25% (6). There are several possible reasons why trained CAPs are reluctant to continue working on mental health for children and adolescents. For instance, children and adolescents with mental disorders usually require less medication, fewer examinations, more frequent hospitalizations and discharges, and more complex doctor-patient relationships. Yet, their total income is usually lower than that of psychiatrists in general hospitals or general psychiatry departments.

### 3.3 Post transfer training for psychiatrists in China

Since 2013, the NHC has encouraged licensed physicians in clinical or traditional Chinese medicine (including traditional Chinese medicine, integrated traditional and Western medicine, and ethnic minority medicine) to add mental health as an additional area of practice. After more than a decade of training, the number of counties in China without mental health resources has significantly decreased (20). NHC Director Lei Haichao stated that from 2013 to 2023, a total of over 16,000 physicians from other specialties completed post transfer training for psychiatrists, with over 10,000 trainees transitioning to work in psychiatry (25). However, fewer than 50% of these physicians ultimately continued to provide long-term psychiatric services (20). Meanwhile, these doctors who received specialized training in psychiatry have no information on what kind of mental health services they provide after returning to their institutions. There is also no data indicating the proportion of the working effort spent by those providing mental health services in providing these services (14).

Despite the rapid growth in the number of psychiatrists, the increase in the number of CAPs has not met expectations. In fact, due to the short duration (1–2 years) of psychiatric training programs across different regions nationwide, as well as the inconsistent content and quality of these programs, the quantity and quality of mental health services that participating physicians can provide are severely limited. A study in Jiangxi Province, China, noted that between 2013 and 2018, a total of 403 physicians completed post transfer training for psychiatrists, accounting for 30.2% of the province’s total number of psychiatrists (26). While this policy has indeed increased the number of practicing (or assistant) psychiatrists, the quantity and quality of mental health service providers require rigorous assessment to avoid a significant imbalance between quantity and quality (14). It is worth noting that currently, in primary-level hospitals (such as county-level, district-level, or township health centers), psychiatrists who have undergone post transfer training are responsible for part of the mental health services for children and adolescents. However, due to factors such as short training duration, inconsistent training quality, and insufficient time dedicated to children and adolescents’ mental health work, they may struggle to meet the actual mental health service needs of this population.

## 4 Proposals

China has a large population of children and adolescents. According to current epidemiological data on mental disorders among minors, approximately 40 million children and adolescents require mental health services. After more than a decade of development, the number of psychiatric medical institutions, psychiatrists, and clinical psychotherapists in China has been gradually increasing. Although the shortage of CAPs is a consensus in the field of psychiatry in China, it is also challenging for the Chinese Medical Doctor Association and the Chinese Psychiatrists Association to determine the exact number of child psychiatrists across the country. Currently, there are no laws or regulations mandating that doctors engaged in the diagnosis and treatment of mental disorders in children and adolescents must hold a specific “child psychiatry” specialist certificate. The lack of a specialized certification system for child psychiatrists in China is mainly caused by multiple reasons, including a relatively short history of professional development, an overall shortage of psychiatrists, insufficient refinement of sub-specialties in the existing certification system, limited professional training resources, and the fact that policies and regulations have not yet imposed mandatory requirements. This situation has led to a series of serious adverse effects, such as inconsistent professional levels of CAPs, increased risks of misdiagnosis and improper treatment, delayed intervention, improper resource allocation, damaged trust, hindered discipline development. It is unable to effectively meet the growing demand for mental health services for children and adolescents.

Currently, we should cultivate more professionals in the field of psychiatry by continuously expanding the enrollment scale of undergraduate programs in psychiatry, strengthening standardized training for resident physicians in psychiatry, promoting and optimizing retraining for psychiatrists, and enhancing continuing medical education training that combines academic education with continuing education. Of particular concern is the need to improve the professional capabilities of doctors in the field of child and adolescent mental health by organizing training programs of various forms and levels, thereby strongly supporting the development of child and adolescent mental health subspecialties.
